# The impact of muscle energy technique and aromatherapy on pain, disability, and sleep quality in office workers with nonspecific neck pain: randomized controlled study

**DOI:** 10.1590/1806-9282.20250942

**Published:** 2026-04-20

**Authors:** Ayça Aytar, Mustafa Gülşen, Selin Özen, Yasemin Kavuncubaşı Genç, Aydan Aytar

**Affiliations:** 1 Başkent University, Vocational School of Health Services, Department of Physiotherapy – Ankara, Turkey.; 2 Yüksek İhtisas University, Faculty of Health Sciences, Department of Occupational Therapy – Ankara, Turkey.; 3 Güven Hospital, Department of Physical Medicine and Rehabilitation – Ankara, Turkey.; 4 University of Health Sciences, Gulhane Faculty of Physiotherapy and Rehabilitation, Department of Orthopedic Physiotherapy and Rehabilitation – Ankara, Turkey.

**Keywords:** Neck pain, Aromatherapy, Lavender oil, Isometric contraction

## Abstract

**OBJECTIVE:**

The aim of this study was to look at the effects of muscular energy technique method and aromatherapy on pain levels and neck disability in office workers with nonspecific neck pain.

**METHODS:**

This study involved 45 women diagnosed with nonspecific neck pain. Participants were randomized into three groups: aromatherapy (n=15), muscle energy technique (n=15), and control (n=15). Outcome measures included Visual Analog Scale for pain, Neck Disability Index, and Pittsburgh Sleep Quality Index. All measurements were conducted before and after the study.

**RESULTS:**

Neck pain, disability, and sleep quality improved in both the aromatherapy and muscle energy technique groups (p<0.001), with no significant between-group difference. In addition, both aromatherapy and muscle energy technique were significantly better at improving neck pain and sleep quality, and reducing neck disability when compared to the control group (p<0.017).

**CONCLUSION:**

This study provides evidence that both aromatherapy and muscle energy technique are safe and practical treatments, which are efficacious in the reduction of pain, disability, and sleep quality in nonspecific neck pain.

## INTRODUCTION

Neck pain (NP) is widely recognized as a significant global public health concern, impacting between 14 and 71% of individuals during their life span^1^. Working with computers for prolonged periods, maintaining the same posture for extended durations, and working continuously can lead to musculoskeletal issues. Studies conducted among office workers have shown that prolonged desk work significantly leads to NP; complaints occurring in the neck and upper extremities among office workers constitute 45% of occupational diseases and can therefore be defined as the most commonly occurring occupational diseases^2^. It has been reported that the prevalence of NP is higher in women than in men, and that hormonal, biomechanical, and psychosocial factors play a role in this difference. Therefore, focusing on NP in female office workers is important to identify risk groups^3,4^.

There are several therapy options available for treating NP, including physical therapy, exercise programs, manual therapy, pharmacological interventions, and ergonomic training. Manual treatment approaches, on the other hand, have grown in popularity in several nations during the last few years^5^. Aromatherapy (AT) massage is another therapeutic intervention used in the treatment of musculoskeletal problems^6^. Muscle energy technique (MET) is a form of manual therapy that utilizes active muscle contraction and relaxation to restore normal muscle length and tone and improve joint mobility, thus improving musculoskeletal mechanics and relieving pain^7^. To date, there is a single study in the literature comparing the benefits of MET and Thai massage therapy on NP, which found that their effects on pain were similar. However, there are no studies comparing the efficacy of MET versus AT massage in the treatment of NP and disability due to NP^8^. The aim of our study was to determine and compare the effects of AT massage and MET on NP, ND, and sleep quality in female office workers with nonspecific NP.

## METHODS

### Population and participants

Between February and September 2024, a group of 45 female office workers with nonspecific diagnoses participated in the study. Cohen's definition of large effect size was used as the basis for determining the sample size required to test the research hypothesis. For the two-way repeated measures analysis of variance, which included between-groups factor (three levels) and repeated measurements over time, an effect size of f=0.40 was assumed, with a 95% confidence level and 85% test power targeted. Accordingly, three groups of 15 participants each were planned, and a total of 45 participants were included in the study. The G-Power 3.1 program was used to determine the minimum number of participants to enter the groups. Those with a history of neck-related surgery, trauma or fracture in the cervical spine, and serious pathologies are excluded from the study. Participants were randomly allocated to AT (intervention) (n=15), MET (intervention) (n=15), and a control group receiving exercise only (control) (n=15) with a 1:1:1 allocation ratio using a computer-based program. The study (CTN: NCT06931314) was approved by Baskent Univ. Ethics (KA24/57) and funded by its Research Fund. The study complied with the Declaration of Helsinki and Türkiye's ethical standards.

The diagnosis of chronic NP was established by a specialist physician in the clinic based on clinical examination and patient history. AT and MET interventions were administered by an experienced physiotherapist in the physical therapy and rehabilitation clinic of the Baskent University Hospital. No serious adverse events were observed during the study; only mild transient muscle soreness was reported by some participants following the sessions.

### Applications

All participants were given home exercises.

#### Aromatherapy group

The AT group was also treated with AT massage 2 days a week for 4 weeks, totaling eight sessions. Participants were made to smell lavender oil before the application, and those who were uncomfortable with the smell were excluded. A massage was applied to the neck area (using Effleurage and petrissage techniques) with lavender oil for 20 min^9^.

#### Muscle energy technique group

All participants did home neck exercises. The MET group received eight sessions over 4 weeks (20 min, 2 days/week). Post-isometric relaxation was applied to the upper trapezius and levator scapula muscles (3–5 reps, 20% max isometric contraction for 7–10 s, stretch for 30–60 s) to address muscular imbalances^10^.

#### Control group

People in the control group were given home exercises including neck isometric, posture, and stretching exercises. They were asked to perform 10 repetitions of each movement twice a day for 4 weeks^11^.

### Evaluations

All measurements were conducted before (week 0) and after treatment (4 weeks later). NP severity was measured with the Visual Analog Scale (VAS). Pain severity was calculated based on this marking (0=no pain, 10=unbearable pain)^12,13^. Neck Disability Index (NDI) was used to assess the severity of neck disability (ND) in individuals participating in the study^14^. The Pittsburgh Sleep Quality Index consists of seven subcomponents measuring subjective sleep quality, sleep latency, sleep duration, sleep efficiency, sleep disturbances, use of sleep medication, and daytime dysfunction^15^.

### Statistical analysis

Data were analyzed using IBM SPSS 25 (IBM Corp., Armonk, NY, USA). Descriptive statistics are presented. Normality was assessed using the Shapiro-Wilk test and histogram. Paired t-tests were used for normally distributed data, and ANOVA was applied for multiple comparison tests. Post hoc pairwise comparisons were performed using the Bonferroni correction to control for increased type I error associated with multiple testing. Accordingly, the significance threshold was set by dividing the alpha level (0.05) by the number of pairwise comparisons, yielding a corrected significance level of p<0.017.

## RESULTS

**Figure 1:**
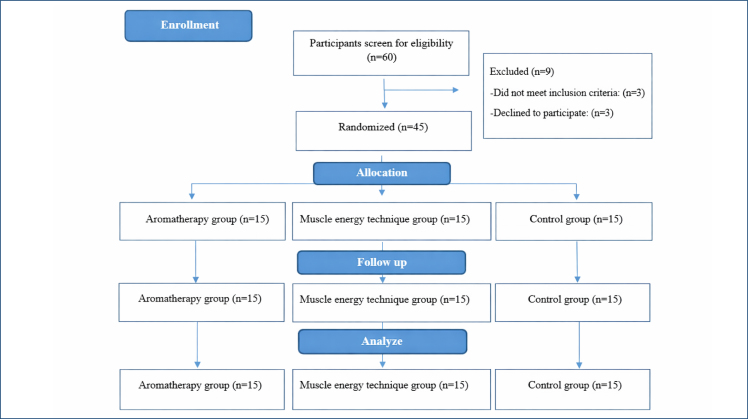
Flowchart of the study.

A total of 45 participants with NP were included in this study (Figure 1). The study participants’ sociodemographic data are presented in Table 1. The comparison of variables within groups is shown in Table 2, and the comparison of aftertreatment outcomes between treatment groups is presented in Table 3. Neck pain at rest and during activity significantly improved in both the AT and MET groups; comparing NP at rest and activity between the AT and MET groups before and aftertreatment revealed a similar effect (p>0.999 and p=0.498, respectively) and both treatments were significantly more effective than exercise alone (p <0.001). When comparing the differences between groups before and aftertreatment for total PSQI, sleep improved in both the AT and MET groups (p>0.999). In both groups, the improvement was significantly more than in the control group (p<0.001). ND improved in both the AT and MET groups to the same degree (p>0.999), and significantly more so when compared to the control group (p<0.001).

**Table 1:** Study participant's sociodemographic data.

		AT group (n=15)	MET group (n=15)	Control group (n=15)	p-value
Age, X (SD)		41.20 (5.12)	42.13 (9.31)	44.00 (6.26)	0.553^a^
Marital status n (%)					
	Married	9 (60)	9 (60)	14 (93.3)	
	Single	6 (40)	6 (40)	1 (6.7)	
Chronic disease n (%)					
	Yes	3 (20)	4 (26.7)	7 (46.7)	
	No	12 (80)	11 (73.3)	8 (53.3)	
Cigarette usage, n (%)					
	Yes	5 (33.3)	6 (40.0)	5 (33.3)	
	No	10 (66.7)	9 (60.0)	10 (66.7)	
Alcohol consumption n (%)					
	Yes	7 (46.7)	7 (46.7)	2 (13.3)	
	No	8 (53.3)	8 (53.3)	13 (86.7)	
Etiology of neck pain, n (%)					
Disk herniation, n (%)		4 (26.7)	1 (6.7)	8 (53.3)	
Straight neck		9 (60)	13 (87.7)	3 (20)	
Cervical dprain		1 (6.7)			
Myofascial pain syndrome		1 (6.7)	1 (6.7)	4 (26.7)	
Pain duration, months, X (SD)		48.80 (41.25)	64.00 (64.56)	44.00 (33.45)	0.501^a^
Work duration, years, X (SD)		15.93 (7.98)	19.00 (11.55)	17.33 (8.37)	0.675^a^

AT: aroma therapy; MET: muscle energy technique, X: mean, SD: standard deviation, n: patient number, %: column percent. Numerical data are given as mean (standard deviation).

a ANOVA.

**Table 2:** The comparison of variables within groups.

		AT group (n=15) X (SD)			MET group (n=15) X (SD)			Control group (n=15) X (SD)			pβ
		Before	After	p	Before	After	p	Before	After	p	
VAS											
	Rest	4.80 (2.56)	1.06 (1.66)	<0.001	3.86 (1.84)	0.46 (1.06)	<0.001	4.26 (1.86)	5.53 (2.19)	<0.001	0.488
	During activity	7.06 (1.83)	2.13 (2.26)	<0.001	5.46 (2.53)	1.06 (1.33)	<0.001	6.66 (2.05)	7.53 (2.44)	0.109	0.120
	PSQI	8.60 (4.23)	4.53 (3.20)	<0.001	7.73 (2.76)	5.33 (2.55)	<0.001	5.40 (2.26)	8.86 (2.66)	0.001	0.025
	NDI	14.80 (6.32)	4.60 (4.17)	<0.001	13.00 (3.81)	5.73 (4.94)	<0.001	9.46 (5.30)	14.93 (7.79)	<0.001	0.026

AT: aromatherapy; MET: muscle energy technique; n: number, X: mean, SD: standard deviation, VAS: visual analogue scale, PSQI: Pittsburg Sleep Quality Index, NDI: The Neck Disability Index. Paired Sample t-test, p<0.05,

β Baseline Group Comparison for homogeneity (p>0.05).

**Table 3:** The comparison of aftertreatment outcomes between treatment groups.

		AT group (n=15) X (SD)	MET group (n=15) X (SD)	Control group (n=15) X (SD)	p	AT group—control group	MET group—control group	AT group—MET group
VAS								
	Rest	1.06 (1.66)	0.46 (1.06)	5.53 (2.19)	<0.001^a^	<0.001^b^	<0.001^b^	>0.999^b^
	During activity	2.13 (2.26)	1.06 (1.33)	7.53 (2.44)	<0.001^a^	<0.001^b^	<0.001^b^	0.498^b^
	PSQI	4.53 (320)	5.33 (2.55)	8.86 (2.66)	<0.001^a^	<0.001^b^	0.004^b^	>0.999^b^
	NDI	4.60 (4.17)	5.73 (4.94)	14.93 (7.79)	<0.001^a^	<0.001^b^	<0.001^b^	>0.999^b^

AT: aromatherapy; MET: muscle energy technique; n: number, X: mean, SD: standard deviation, VAS: visual analogue scale, PSQI: Pittsburg Sleep Quality Index, NDI: The Neck Disability Index.

a ANOVA,

b Bonferroni correction p<0.017.

## DISCUSSION

To the best of the researchers’ knowledge, this is the first study comparing the effects of AT and MET on office workers with nonspecific NP. According to the findings of this study, both AT and MET improve symptoms of NP, ND, and sleep quality with no evidence of superiority of one technique over the other. In addition, the findings provide evidence that both AT and MET improve these parameters significantly more than exercise alone. In fact, exercise alone increased NP and disability and reduced sleep quality. In this study, the findings of improved NP and ND with massage and MET are similar to previous studies. The lack of structured supervision in the control group—who were only provided with general exercise advice and performed home-based exercises without monitoring—likely contributed to poor adherence. Previous studies have shown that unsupervised home exercise programs are often associated with low compliance and limited effectiveness^16^. Buttagat et al. found that Thai massage and MET significantly decreased NP, and improved ND and neck range of motion in individuals with chronic neck pain, with no outcome difference between the two techniques^8^. Similar positive outcomes regarding pain and range of motion following massage have also been reported in chronic upper and lower back pain^8,17^. The efficacy of aromatic essential oils, including lavender, in the reduction of NP and ND in comparison to a placebo ointment has previously been shown^18^. Essential oils such as lavender and marjoram have been shown to significantly reduce pain and depression in patients with arthritis and cancer^19^. Moreover, many studies have reported that massage with lavender oil can reduce perception of muscle pain^20,21^. Aromatherapy is thought to stimulate the parasympathetic nervous system, thus aiding relaxation and inducing pleasant sensations^22^. A study comparing AT massage in nonspecific NP in combination with acupoint electrode stimulation to conventional treatment alone also reported reduced NP, stiffness, and stress and increased range of motion in the intervention group. The findings of both this study and past studies suggest that AT is a reasonable complementary treatment of choice, which can accompany conventional treatments in NP^21^. In recent years, the MET has grown in popularity as a manual therapy in the treatment of musculoskeletal pain, and research into its efficacy has grown in parallel to this. Even though the precise mechanism of MET-induced pain relief remains unknown, it is believed that MET acts on joint proprioceptors and mechanoreceptors, causing an effect on descending pathways and resulting in a change in the motor programming of joint^23^. It is also believed that MET changes the viscoelastic properties of the soft tissues, improving flexibility and increasing stretch tolerance^24,25^. A systematic review on the efficacy of the MET included three studies in which treatment of NP and ND using MET was compared to exercise, stretching, and a mobilization intervention. In all three studies, MET was found to give superior results in both outcome parameters^26^.

In the study by Qin et al., it was summarized that the minimally significant change for the PSQI was 3 points, and the MCID was reported to be in the range of ≈2.5–2.7^27^. In our study, the PSQI improvement in the AT group exceeded the MCID value, and the significant and clinically beneficial decrease in this score, which decreased the PSQI score below the cutoff point of 5, led to a change in our patient profile (from 8.60 to 4.53), thus indicating a clinical class change^28^.

The improvements in sleep quality are believed to occur due to massage and MET stimulating the blood supply to the muscles, hence muscle oxygenation, relaxation, and increased joint mobility. Other bodily responses include improvement in posture, well-being, and movement patterns. All of these factors contribute to pain reduction. In addition, similar to the findings in this study, a study comparing the effects of Swedish massage versus MET on sleep quality measured using the PSQI revealed that both treatments significantly improved sleep disturbance. However, there was improvement in all PSQI components in the MET group, whereas there were improvements in only four components of the index in the massage group^29^.

AT massage and MET, used for muscle relaxation to reduce NP, are among the noninvasive complementary treatment methods and are generally considered to have a high safety profile. The risks of gastrointestinal side effects, drug interactions, or systemic complications likely seen with pharmacological treatments are quite low with AT massage and MET. Similarly, serious complications such as infection, nerve, or vascular injury, which can be seen with invasive procedures, are reported to be virtually nonexistent with these methods. Furthermore, MET, which offers a gentler and more controlled approach compared to high-speed manipulation techniques, is considered safer for neck and spine health. However, it is emphasized that both methods may have mild side effects, such as allergic reactions, muscle tenderness, or short-term dizziness, and that contraindications should always be considered^30,31^.

## CONCLUSION

This study provides evidence that both AT and MET are safe, practical, and inexpensive treatments, which are efficacious in the reduction of pain and disability in nonspecific NP and may also have a positive effect on sleep quality. Further studies are needed to investigate the short-, medium-, and long-term effects of AT and MET in the treatment of NP in order to substantiate these positive findings.

### Clinical implications

The present findings suggest that AT and MET can serve as complementary treatment strategies for patients with NP, particularly in improving pain and sleep quality. For home-based exercise programs, supervised implementation is strongly advised, which may be supported through exercise diaries or remote monitoring systems to enhance adherence and ensure patient safety.

### Limitations

This study has limitations that need to be addressed. Our sample consisted solely of women with NP, and we are unsure whether these results can be generalized to men. Another limitation of our study is the lack of remote monitoring of the control group's home exercises. Therefore, we recommend further research on this topic.

## Data Availability

The datasets generated and/or analyzed during the current study are available from the corresponding author upon reasonable request.
